# Supplementary material for: A Chronological Study on Grapevine Leafroll-Associated Virus 2 in Australia
Source: Viruses. 2023 Apr 30;15(5):1105. doi: 10.3390/v15051105 (PMC10223255; doi:10.3390/v15051105)
Supplement: Supplementary file 1 [file viruses-15-01105-s001.zip › viruses-2272705-supplementary.pdf]

Supplementary Materials

The following Supplementary Materials are available online at https://www.mdpi.com/1999-491 5/13/1/131/s1,

Table S1. Primers used in this study to detect grapevine viruses using RT-PCR

Virus[1]	Primer name	Sequence	Amplicon size (nt)	Target gene	T_a[3]	Ref
GLRaV-1	p35LR17589f	AATCCTATGCGTCAGTATGC				
	p35LR17763r	TGGCATCGTTGCTAAATTGAG	174	CP	56	up[4]
GLRaV-2	LR2-U2	ATAATTCGGCGTACATCCCCACTT				
	LR2-L2	GCCCTCCGCGCAACTAATGACAG	332	HSP	60	[19]
	V2dCPf2	ACGGTGTGCTATAGTGCGTG				
	V2CPr1	GCAGCTAAGTACGAATCTTC	515	CP	56	[19]
GLRaV-2-RG[2]	RGHSP227V	GCGACTCCAGCAACTTTAGTGA				
	RGHSP777C	GTCTAACGAAAGATCGGGTTCTAAG	550	HSP	56	[40]
GLRAV-3	LR3P-H420	GATTTAAGCGCGTTTTTCAGGAC				
	LR3P-C629	CGGCACGATCGTACTTTCTAA	210	p55	57	[47]
GLRaV-4	HSPLR4-F	CTCAAACCAGCGGCTGTTG				
	HSPLR4-R	GTGATACCATATACATACCGACC	441	HSP	55	[48]
GLRaV-4/6	LR6CPF	AAATTCCGCGCCTMTTCAATG				
	LR6CPR	ATGTCTGGGGCCACCTTAGT	563	CP	57	up
GLRaV-4/9	LR9F	ACAGTGGTCGGCATAAGAAAAG				
	LR9R	ACACAAACATGCAGGCCAAAG	250	HSP70h	57	[49]
GRSPaV	RSP48	AGCTGGGATTATAAGGGAGGT				
	RSP49	CCAGCCGTTCCACCACTAAT	330	CP	56	[50]
GVA	GVA-H7038	AGGTCCACGTTTGCTAAG				
	GVA-C7273	CATCGTCTGAGGTTTCTACTAT	236	RNA binding	55	[51]
GFkV	GFkV-U279	GGTCCTCGGCCCAGTGAAAAAGTA				

	GFkV-L630	GGCCAGGTTGTAGTCGGTGTTGTC	352		60	[52]
GVB	GVB-H6980	GTGCTAAGAACGTCTTCACAGC				
	GVB-C7439	ATCAGCAAACACGCTTGAACCG	460	RNA binding	60	[51]
GPGV	GPGVCP167F	GCCTTTGCAATTGATGCCCA				
	GPGVCP167R	ACCGACTTTTGATTTGGGG	167	CP	54	up

[1] GLRaV, Grapevine leafroll-associated viruses; GRSPaV, grapevine rupestris stem pitting-associated virus, Grapevine virus A (GVA); GFkV, Grapevine fleck virus; GPGV, Grapevine pinot gris virus. [2] Specific primers used to detect RG phylogroup of GLRaV-2. [3] Annealing temperature [4] Unpublished.

Table S2. Basic statistics of grapevine leafroll-associated virus 2 contigs of two Grenache grapevines used in the phylogenetic analysis.

Vine No.	Raw reads	Trimmed reads	No. of GLRaV-2 contigs	Acc No.	Isolate name/ Contig ID	Contig length	Average coverage	Mapped reads
Grenache 2	8513956	8470781	11	OP743924	GrenV2N10154PN	897	433.697	2863
				OM220045	GrenV2N60H4	6330	281.655	13349
				OM220043	GrenV2N5102PN	1463	282.887	3108
				OM179872	GrenV2N20	16467	172.452	20826
				NA[1]	GrenV2N12	10455	341.479	28790
				NA	GrenV2N40	7278	339.714	19759
				NA	GrenV2N931	2995	186.39	4428
				NA	GrenV2N4854	1507	181.155	2173
				NA	GrenV2N5770	1364	270.349	2904
				NA	GrenV2N10829	862	295.883	2105
				NA	GrenV2N38211	305	350.148	849
Grenache 5	7949612	7912921	14	OK324337	GrenV5N5	16476	306.903	34284
				OM362846	GrenV5N6H4	16517	370.556	42047

	OP743925	GrenV5N10473	857	753.886	4389
	NA	GrenV5N720	3279	393.404	9787
	NA	GrenV5N1335	2645	508.319	10145
	NA	GrenV5N4487	1590	693.301	8359
	NA	GrenV5N11393	845	566.553	3639
	NA	GrenV5N16970	583	906.772	4051
	NA	GrenV5N26382	405	732.326	2231
	NA	GrenV5N29816	371	365.887	1003
	NA	GrenV5N2950	1939	368.15	5404
	NA	GrenV5N3937	1702	345.687	4456
	NA	GrenV5N6564	1268	553.856	5313
	NA	GrenV5N7584	1160	1010.523	9007

[1] GLRaV-2 contigs that were not used in phylogenetic analysis of this study.

Table S3. Details of grapevine leafroll-associated virus 2 isolates used for phylogenetic, and recombination analysis.

Acc No.	Isolate	Phylogroup	Country	Variety	Type of analysis[3]
OM220043	GrenV2N5102	PN	Australia[1]	Grenache	CP
OM220045	GrenV2N60	H4	Australia[1]	Grenache	CP, p24
OM362846*	GrenV5N6	H4	Australia[1]	Grenache	CP, p24, FG
OM179872*	GrenV2N20	93/955	Australia[1]	Grenache	CP, p24, FG
OK324337*	GrenV5N5	93/955	Australia[1]	Grenache	CP, p24, FG
OK334632	Chard-SA	PN	Australia[2]	Grenache	CP
OK334633	Chard-Vic	PN	Australia[2]	Grenache	CP
OK334631	RG	RG	Australia[2]	Grenache	CP
OP743924	GrenV2N10154	PN	Australia[1]	Grenache	p24

OP743925	GrenV5N10473	PN	Australia[1]	Grenache	p24
KX774192*	ISA-BR	H4	Brazil	Vitis labrusca cv. Isabel	CP, p24, FG,
MH814502*	14G466	PN	Canada	*Vitis vinifera*[4]	CP, p24
NC004724	GRSLaV	RG	USA	Red Globe	FG
MH814500*	12G4102	RG	Canada	*Vitis vinifera*	CP
KU508672*	Goldfinger	RG	China	Goldfinger	CP, p24, FG
EF012718	PV44	BD	France	Chardonnay	CP
EF012721	PV20	PV20	France	Savagnin	CP
EF012717	PV124	PV20	France	Negro Amaro	CP
AY697863	H4	H4	Italy	*Vitis vinifera*	CP
DQ286725*	BD	BD	Italy	Don Mariano	CP, p24, FG
MN548394*	SK933	RG	Slovakia	*Vitis vinifera*	CP, p24, FG
AY881628*	93/955	93/955	South Africa	*Vitis vinifera*	CP, p24, FG
MW715830	31004	PN	Spain	Tempranillo	CP, p24
JQ771955*	OR2	PN	USA	Pinot Noir	CP, p24, FG
AF314061*	GRSL	RG	USA	*Vitis vinifera*	CP, p24, FG
KF220376*	SG	H4	USA	Sangiovese	CP, p24, FG
MW715828	29085	93/955	Spain	Malfar	p24
FJ436234*	OR1	PN	USA	Vitis vinifera	FG
JX559644*	3138-07	PN	Canada	Vitis vinifera	FG
MH814497*	12G410	93/955	Canada	Vitis vinifera	FG
KY821090*	SL13	H4	USA	Bai Ji Xin	FG
KY821091*	SL45-2	H4	USA	Zi de qv	FG
KY821092	SL53	H4	USA	cui fei me	FG
MH814493*	12G402A	H4	Canada	*Vitis vinifera*	FG
MH814496*	12G407B	H4	Canada	*Vitis vinifera*	FG

JX513891*	1050-02	RG	Canada	*Vitis vinifera*	FG
MT899926*	DSJPN2	RG	Canada	Pinot Noir	FG
MH814495*	12G407	BD	Canada	*Vitis vinifera*	FG
MH814494*	12G404	BD	Canada	*Vitis vinifera*	FG
MH814492*	12G402B	BD	Canada	*Vitis vinifera*	FG

[1] The Australian isolates sequenced by HTS. [2] The Australian isolates sequenced by Sanger sequencing. [3] CP = phylogenetic analysis of coat protein gene, p24 = phylogenetic analysis of p24 gene, FG = phylogenetic analysis of full genome. [4] Variety is not known.
*Complete or near complete genome sequences used for the recombination analysis.
